# Callous-unemotional traits in Chinese preschool children with attention-deficit/hyperactivity disorder

**DOI:** 10.1186/s13034-021-00388-0

**Published:** 2021-07-10

**Authors:** Jinsong Zhang, Wei Li, Huifeng Zhang, Amanda Wilson, Lan Shuai, Weiping Xia, Zhouye Wang, Meihui Qiu, Yuanyuan Wang

**Affiliations:** 1 Department of Medical Psychology, Xinhua Hospital Affiliated to Shanghai Jiao Tong University School of Medicine, 1665 Kongjiang Road, Shanghai, 200092 China; 2 Ministry of Education and Shanghai Key Laboratory of Children’s Environment Health, 1665 Kongjiang Road, Shanghai, 200092 China; 3 Department of Psychology, Faculty of Health and Life Sciences, De Montfort University, Leicester, UK

**Keywords:** Callous-unemotional traits, Attention-deficit hyperactivity-disorder, Conduct problems, Chinese preschool children

## Abstract

**Background:**

Children with early onset of Callous-Unemotional (CU) traits are at a higher risk for long-term, persistent psychosocial problems. The current study aimed to explore the characteristics of CU in preschool children with Attention Deficit Hyperactivity Disorder (ADHD) and the diagnostic significance of CU traits in ADHD.

**Methods:**

A total of 176 preschool children (89 with ADHD and 87 Typically Developing Children [TDC]) aged 4–5 years old were recruited to the study. The participants were assessed for CU traits, emotional and behavioral problems, and how their executive functioning was associated with ADHD using multiple assessment scales. Multiple linear regression analysis was performed to assess the incremental validity of the Inventory of Callous-Unemotional Traits (ICU), adjusting for possible covariates by child’s sex, conduct problems, and oppositional defiant symptoms.

**Results:**

The results showed that there was a significant difference of ICU scores between the ADHD and TDC groups (*F* = 30.12, *P* < 0.001). In terms of callousness, ADHD + Oppositional Defiant Disorder (ODD) group showed a significant high score, and the ADHD only group scored significantly higher than the TDC group (*F* = 20.42, *P* < 0.001). The ICU was negatively associated with the prosocial behaviour subscale (γ = − 0.57, *P* < 0.01) and showed low to moderate positive correlations with emotional and behavioural problems, as well as executive function (γ = 0.24–0.67, *P* < 0.05). The ICU scores explained 6% of the incremental validity in ADHD symptoms. The diagnostic value of the ICU for ADHD was medium and acceptable.

**Conclusions:**

The current study indicated that early identification of CU traits may help clinicians better understand symptoms and behavioural problems in children with ADHD. CU traits therefore could be considered as a useful assessment tool for ADHD.

## Introduction

Callous can be defined as lacking empathy and having a shallow affect [1], which is emphasised within Conduct Disorder (CD). Based on the fifth edition of the Diagnostic and Statistical Manual of Mental Disorders (DSM-5), the diagnosis of CD specifies there is limited prosocial emotions displayed for at least two of the four characteristics, which are lack of remorse or guilt, callous-lack of empathy, unconcerned about performance, and shallow or deficient affect [2]. These four charactersistics are closely combined in children to reflect different personality traits of a person including prosocial emotions. The concept of Callous-Unemotional (CU) traits, as proposed by Frick, refers to low levels of guilt, empathy, and caring for others and is considered to be an early life developmental precursor to latter adulthood psychopathy [3]. It is more generalized to indicate the lacking of prosocial emotions, not just in relation to CD.

Attention-Deficit Hyperactivity Disorder (ADHD) is one of the most common psychiatric disorders, affecting approximately 5% of children and adolescents around the world [4]. Oppositional Defiant Disorder (ODD) and CD frequently occur in comorbidity with ADHD [5], with the prevelance estimated to be between 20%, and slightly more than 40% among preschool children [2]. Across the life span development, in both clinical and community samples, CU traits are closely related to Externalizing Behavior Problems (EBPs) such as ADHD, ODD, CD, and antisocial personality disorder [6–9]. Furthermore, CU traits act as early-emerging characteristics that may be a risk factor for later externalized psychopathology. It is proposed that CU traits are related to the etiology, developing from ADHD to ODD/CD.

As mentioned it is well-documented that CU traits can increase the risk of antisocial behaviour and other psychopathy, such as ODD and CD [10, 11]. Importantly, a review has examined CU as an important characteristic for identifying more aggressive, severe, and pervasive patterns of antisocial behaviour in children older than five and adolescents [12]. Previous studies on CU traits have mainly focused on middle and late childhood and adolescences [13–16], while little attention has been paid to early childhood, especially preschool years where early interventions can be more effective [17]. Research has confirmed that without intervention people with CU traits will have poor outcomes throughout their life [18]. It is therefore necessary to detect CU traits as they can be early indicators of the onset of ODD and CD.

Studies in this area have found that adolescents who have CD with co-occurring CU traits display elevated levels of emotional and behavioural dysregulation [19]. Nothing is known, however, about the CU traits of preschool children with ADHD and the relationship between CU traits and emotional and behavioral problems. It is important to understand the internal links between these behaviors in the preschool period, as this is the time when these behaviors are first emerging, and are most receptive to intervention [20]. To understand the high rates of ADHD co-occurring with ODD/CD, and the earlier onset age of ADHD when compared to ODD/CD, more evidence is needed to make clear whether CU traits are significant in ADHD children in preschool years.

Looking beyond examining CU traits and emotional and behavioral problems, researchers also need to pay close attention to the role of other salient factors in early childhood that are linked to ADHD across development. In particular, executive function (EF), a construct that contains cognitive flexibility, working memory, inhibitory control, and emotional control for the purposes of planning and executing goal-directed activity [21–23], is critical to cognitive and social development [24]. Children with EBPs, including ADHD, are more likely to exhibit EF deficit compared with typically developing children (TDC) [25–27]. Fewer studies have examined the specific link between EF and CU behaviors and even fewer have looked at preschool children. A meta-analysis examining the CU traits within late-adolescents and adults indicated that higher CU traits were associated with less EF deficits [28]. However, the findings for the association between CU and EF in preschool studies were contradictory [20, 29, 30]. Further analysis showed that higher levels of CU behaviors and Conduct Problems (CPs) display relatively better EF in preschool children with EBPs [30]. Therefore, it remains unclear within a preschool ADHD sample the link between EF and CU traits. This study therefore begins to explore the correlations between CU traits, EBPs, and EF.

Due to the early onset of ADHD and the high co-morbidity of ODD/CD, CU traits should be considered as one of the dimensions when evaluating preschool children with ADHD. Children with ADHD often exhibit external and internal behavioural problems, and EF deficits. Empirical data support these findings, as children who experience higher levels of CU traits show an increase in their conduct problems over time and they are more likely to develop ODD or CD [31]. Thus, it is necessary to assess characteristics of CU in early childhood in order to create reliable and valid measures as an indicator for non-drug interventions. Although the commibity of ADHD with ODD/CD is high and common amongst ODD children with high CU traits, it is not clear, especially in preschool children, about the relationship between ADHD symptoms (inattention and hyperactive-impulsive) and CU traits.

Taken together, the aims of the current study are: (1) To determine whether CU traits can be used to discriminate ADHD from TDC, as well as to identify any differences in the characteristics of CU between ADHD and ADHD + ODD; (2) To examine the extent to which EF and EBPs are associated with CU traits; (3) To assess incremental validity of the ICU scale for predicting ADHD symptoms and the specificity and sensitivity of the ICU in the diagnosis of ADHD. The researchers hypothesized that preschool children with ADHD would have higher levels of CU traits than TDC, and the researchers expected ICU to achieve incremental validity in the prediction of ADHD after adjusting for covariates. These three aims will help determine if the ICU could be specific and sensitive for ADHD diagnosis in preschool children.

## Methods

### Participants and recruitment

A total of 89 participants with ADHD and 87 TDC were included in this study. The clinical diagnosis interviews were conducted by psychiatrists based on the Diagnostic Infant Preschool Assessment (DIPA) [2, 4]. In the ADHD group, sixty-two percent of children met the DSM-5 criteria for ADHD alone and thirty-eight percent met ADHD + ODD. All children met the following inclusion criteria: (1) the child’s age range was in the preschool stage from 4 to 5 years and 11 months; (2) children had a full intelligence quotient (FIQ) measured by the Wechsler Primary and Preschool Scale of Intelligence (WPPSI) of ≥ 80; (3) both the child and his or her parents consented to take part in the study; and (4) the child had taken no medication(s) or underwent other behavioural interventions at least 1 week before the tests. Children were excluded from the study if they suffered other severe psychiatric disorders, such as anxiety disorders and mood disorders, or physical health problem, such as epilepsy and traumatic brain injury, that might interfere with the assessment and results.

The current study was conducted in accordance with the Declaration of Helsinki and was approved by the Ethics Committee of Xinhua Hospital affiliated with Shanghai Jiao Tong University (Approval Number: XHEC-C-2014-082). Parental written informed consent (only one parent needed to sign the written informed consent), child assent, and school agreement was obtained before children could participate in the study. All preschool participants’ parents and the school provided written informed consent and the children provided verbal assent. Similarly a written information sheet was provided to parents, the school, and verbal information using language that 4–5 year olds could understand, was provided to the children before consent and assent were obtained.

The two groups, ADHD and TDC participated in the study from October 2016–May 2018 and there were different approaches to recruitment. With the ADHD group the participating preschool children and their parents were recruited from the outpatient clinic in the Department of Clinical Psychology, Xinhua Hospital affiliated with Shanghai Jiao Tong University. Children included in the ADHD group met the criteria for ADHD based on a clinical diagnosis with the DSM-5. The TDC group consisted of 87 healthy control children within the same age range. They were recruited from two kindergartens in the urban areas of Shanghai. The children in the TDC group were excluded if they met the diagnostic criteria for ADHD or other psychiatric disorders.

### Measurements

#### Diagnostic interview

*Diagnostic Infant and Preschool Assessment *(*DIPA*)*.* The DIPA (version 2/28/14) was developed and updated by Scheeringa according to DSM-5 in 2014 [32]. The DIPA is a semi-structured instrument that has been adapted for 13 psychiatric disorders, one of them being ADHD. It is intended as an interview for caregivers of children 6 years and younger. The parents in this study were interviewed using DIPA, according to the DSM-5 diagnostic criteria [2]. The criteria for ODD requires four or more, out of eight, oppositional defiant symptoms. In addition, symptoms must be maladaptive and inconsistent with developmental level, as well as cause impairment. It has been found to be a reliable and valid screening instrument for childhood neurodevelopmental disorders [32].

#### Rating scales

*Swanson, Nolan, and Pelham Rating Scale-IV *(*SNAP-IV*) [33] was used to measure these verity of ADHD symptoms according to inattention symptoms, hyperactive/ impulsive symptoms, and oppositional defiant symptoms. The total score is summed from the three subscale, the higher the score, the more severe the symptoms. The SNAP-IV has relatively good reliability and validity in Chinese preschool children and is well accepted, this was also reported in a previous study by the researchers [34].

*Inventory of Callous-Unemotional Traits *(*ICU*) [35] includes 24 items, and each item is answered on a 4-point Likert scale from 0 (not at all true) to 3 (definitely true), for a total score of 0–72. The inventory assesses CU traits using the parent report version. The ICU has three subscales: Callousness (10 items), Uncaring (nine items), and Unemotional (five items), from which a total score is calculated. It was shown (2013) [29] that the ICU is a promising questionnaire to identify CU traits early in the preschool years.

*Behavior Rating Scale of Executive Function-Preschool Version *(*BRIEF-P*) [36] parent form is a 63 item questionnaire for parents to assess the components of EF in preschool children aged 2–5 years. Each item is rated as one (never), two (sometimes), or three (often). The measure includes five empirically derived clinical scales: inhibition, shifting, working memory, emotional control, and planning/organization. The higher the score is, the lower the EF performance. The BRIEF‑P has been shown to have adequate concurrent and discriminant validity and high reliability in Chinese children [25].

The parent-rated *Strengths and Difficulties Questionnaire *(*SDQ*) [37] was used to measure psychiatric symptoms in children four to seventeen years old. The SDQ comprises three psychiatric subscales, namely, hyperactivity, conduct and emotional problems, along with additional subscales of peer-relationship problems and prosocial behaviour. The higher the score is, the higher the difficulty is; except for the prosocial behaviour subscale. The SDQ (Chinese version) has been widely used with Chinese children. Satisfactory internal consistency and construct validity have been reported for the Chinese version of the SDQ [38].

#### Study procedure

The medical histories of the TDC children were briefly reported by their teachers and parents to exclude children with obvious medical and developmental problems. The children in the case groups and their parents were interviewed in the clinic, and primary diagnoses were made by psychiatrists according to a comprehensive medical history evaluation and psychiatric examination. Informed consent/assent was obtained before conducting any of the assessments. Parents completed questionnaires. The final diagnosis followed the criteria of the DSM-5 by DIPA.

#### Statistical analyses

The data was analysed using SPSS Statistics version 22 (IBM; Armonk, New York, USA) and EpiData3.1 (The EpiData Association, Odense, Denmark). To characterize the sample, frequencies, correlations, means, and standard deviations (SD) were calculated for the variables of interest. Statistical differences in the variables were examined by Analysis of Variance (ANOVA) for continuous variables and Pearson’s chi-square tests for categorical measures. Pearson’s correlations were also used to examine the association between the CU traits, EBPs, and EF. Furthermore, multiple linear regression analysis was performed to assess the incremental validity of the ICU, adjusting for possible covariates by child’s sex, CPs, and oppositional defiant symptoms. Before that, possible covariates were selected based on the previous studies and a correlation analysis was conducted. Finally, the specificity and sensitivity analyses of the ICU was performed using a receiver operating characteristic (ROC) curve. Non-parametric estimates of the area under the curve (AUC) from the ROC curve analyses quantified the diagnostic efficiency of the ICU scale scores. All tests were two-tailed and *P* > 0.05.

## Results

### Descriptive statistics

A total of 176 four- to five-year-old preschool children (89 with ADHD and 87 TDC) were enrolled in this study. The demographic and clinical characteristics of the ADHD group (including ADHD only and ADHD + ODD) and TDC group are presented in Appendix 1. The preschool children with a comorbid ADHD and ODD accounted for 38% of the total sample with ADHD. There was no significant difference of age (*F* = 1.37, *P* = 0.26), sex (*χ*^2^ = 3.39, *P* = 0.18), and IQ (*F* = 1.59, *P* = 0.21) among the three groups (*P* > 0.05).

### Comparisons of CU traits among the ADHD only, ADHD + ODD and TDC groups

The comparison results of the ICU subscale scores among the ADHD only, ADHD + ODD, and TDC groups were analysed by one-way ANOVAs and post hoc tests, as presented in Table 1. The callousness scores in the ADHD + ODD group were significantly higher than those in the ADHD only group *(P* < 0.001) and they were statistically significant after controlling for the child’s age and sex. However, there were no significant differences between children with ADHD + ODD and children with ADHD only in the uncaring and unemotional scores.

**Table 1:** Comparison of CU traits among the ADHD only, ADHD + ODD and TDC groups (mean ± SD)

Subscales	ADHD only (*n* = 55)	ADHD + ODD (*n* = 34)	TDC (*n* = 87)	*F*	Post hoc test
Callousness	8.67 ± 5.29	10.82 ± 4.83	5.45 ± 3.58	20.42***	ADHD + ODD > ADHD > TDC
Uncaring	15.87 ± 4.03	16.82 ± 3.75	10.79 ± 4.07	40.60***	ADHD = ADHD + ODD > TDC
Unemotional	4.83 ± 2.45	4.18 ± 2.39	4.23 ± 2.34	1.27	n.s.d
Total ICU	29.70 ± 10.4	32.04 ± 8.30	21.39 ± 7.40	30.12***	ADHD = ADHD + ODD > TDC

***P < 0.001

*n.s.d.* not significant difference, *CU* callous-unemotional, *ADHD* *only* ADHD alone, *ODD* oppositional defiant disorder, *ADHD + ODD* ADHD and comorbid ODD, *TDC* typically developing child, *ICU* inventory of callous-emotional traits

### Correlations of CU traits with emotional and behavioural problems and executive function

As seen in Table 2, after accounting for the child’s age, sex, and child IQ, Pearson’s correlation analyses indicated that there was a significant correlation between the ICU, emotional and behavioural problems, and EF. In terms of EBP variables, ICU was positively associated with emotional problems (γ = 0.24, *P* = 0.02), CP (γ = 0.52, *P* < 0.01) and peer problems (γ = 0.47, *P* < 0.01), and negatively associated with prosocial behaviours (γ = 0.57, *P* < 0.01). ICU was positively associated with EF performance (γ = 0.67, *P* < 0.01).

**Table 2:** Pearson’s correlations coefficients between variables

Variable	1	2	3	4	5	6
1. CU( ICU)	-					
2. EPs(SDQ)	0.24*	-				
3. CPs(SDQ)	0.52**	0.25*	-			
4. PPs(SDQ)	0.47**	0.27**	0.41**	-		
5. PBs(SDQ)	− 0.57**	− 0.21*	− 0.43**	− 0.39**	-	
6. EF(BRIEF-P)	0.67**	0.32**	0.46**	0.54**	− 0.42**	-

*P < 0.05, **P < 0.01

*CU* callous-unemotional traits, *ICU* inventory of callous-unemotional traits, *SDQ* strengths and difficulties questionnaire, *EPs* emotional Problems, *CPs* conduct problems, *PPs* peer problems, *PBs* prosocial behaviours, *EF* executive function, *BRIEF-P* behavior rating scale of executive function-preschool version

### The influence of CU traits on ADHD assessment

Linear regressions were performed to assess the association of CU traits and oppositional defiant and ADHD symptoms (inattention and hyperactive/ impulsive). In the linear regressions, the correlations were all significant, with inattention symptoms having higher beta values (*β* = 0.55, *R*^2^ = 0.20, *P* < 0.001) than hyperactivity symptoms (*β* = 0.39, *R*^2^ = 0.15, P < 0.001). The CU traits were also associated to oppositional defiant symptoms (*β* = 0.32, *R*^2^ = 0.11, *P* < 0.001). The ICU score was significantly correlated with ADHD symptoms and explained a proportion (15–24%) of the variance, as shown in Table 3. Binary logistic regression analysis was performed to assess the influence of CU traits on ADHD diagnosis (is or not). The odds ratio (OR) value of ICU score was 1.15, 95% CI = 1.10–1.20, P < 0.05, which suggested that the CU traits were associated with poor performance due to ADHD.

**Table 3:** Linear regression analysis for the effect of CU traits on the symptoms of ADHD

Variable	*β*	*R*^*2*^	*t*	*P*
IA(SNAP-IV)	0.55	0.20	8.704	< 0.001
H/I(SNAP-IV)	0.39	0.15	5.224	< 0.001
ADHD symptoms	0.49	0.24	7.023	< 0.001
ODD symptoms (SNAP-IV)	0.32	0.11	4.264	< 0.001

*IA* in attention symptom, *H/I* hyperactive/impulsive symptom, *ODD* oppositional defiant disorder, *β* standardized regression coefficient, *R*^*2*^ determination coefficient, *CU* callous-unemotional traits, *ADHD* attention-deficit/hyperactivity disorder

### Covariates

Given the correlation between CU traits and CPs and oppositional defiant symptom, the relationship between ICU factors and ADHD symptoms may be affected by confounding factors such as CPs and oppositional defiant symptom. Taking age, sex, IQ, CPs, and oppositional defiant symptoms as independent variables, and CU traits and ADHD symptoms as dependent variables, the results of the single linear regression models showed that sex, CPs, and oppositional defiant symptoms were significantly related to CU traits (*β* = − 0.16, 0.50, 0.34, respectively, *P* < 0.05) and ADHD symptoms (*β* = − 0.22, 0.34, 0.65, respectively, *P* < 0.01), as shown in Appendix 2. The covariates included child sex, CPs, and oppositional defiant symptoms due to their correlations with CU traits and ADHD symptoms.

### Incremental validity of the ICU scale for predicting ADHD symptoms

Multiple linear regression analysis was performed to assess the incremental validity of the ICU, as shown in Table 4. The results indicated that sex (*β* = − 0.13, *P* < 0.05), oppositional defiant symptoms *(β* = 0.55, *P* < 0.001), and CU traits (*β* = 0.27, *P* < 0.001) predicted the presence of ADHD symptoms. When the influence of sex and oppositional defiant symptoms were controlled, the analysis results showed that ICU could be used to explain 6% of the incremental validity in ADHD symptoms.

**Table 4:** Multiple linear regression analysis for the incremental validity of the ICU

	ADHD symptoms		
	*β*	*T*	*P*
Model 1			
Sex	− 0.17	− 2.85	0.005
OD	0.64	11.04	0.00
*R1*^*2*^	0.45		
Model 2			
Sex	− 0.13	− 2.38	0.02
OD	0.55	9.37	0.00
CU	0.27	4.51	0.00
*R2*^*2*^	0.51		
*ΔR*^*2*^	0.06		

The standardized regression coefficients (*β*) were calculated by converting all predictors and outcomes to z-scores before the analysis

Model 1 predictive variable: sex, OD

Model 2 predictive variable: sex, OD, CU

*β* standardized regression coefficient, *OD* oppositional defiant, *CU* callous-unemotional, *ADHD* attention-deficit/hyperactivity disorder

### Specificity and sensitivity of the ICU scale

**Fig. 1:**
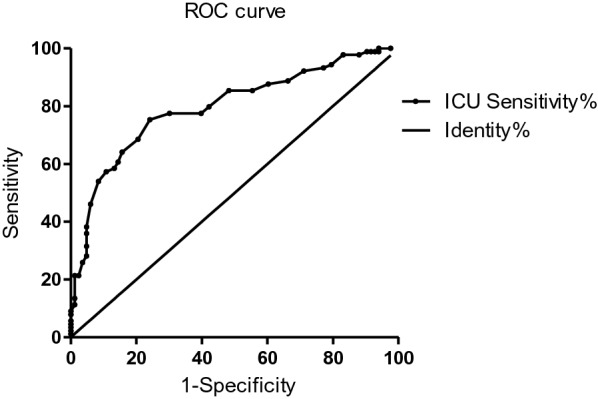
ROC curve of the ICU questionnaire. *AUC* area under the curve, *CI* confidence interval

The ROC was calculated to analyse the specificity and sensitivity of the ICU scale in the diagnosis of ADHD, and to differentiate the ADHD group from the TDC group. According to the ROC curve, shown in Fig. 1, the tangent point with the largest Youden index nearest to the upper left, was selected as the key critical point; that is, when the ICU total score was 24.5, the sensitivity of the diagnosis of ADHD was 0.753, and the specificity was 0.759 (AUC = 0.795, 95% CI = 0.67–0.84, *P* < 0.001).

## Discussion

The current study focuses on the importance of CU traits. Using the ICU, the results of our study showed that CU traits in Chinese preschool children with ADHD only and ADHD + ODD were significantly higher when compared to those children in the TDC group. This study is, to our knowledge, the first to examine the characteristics of CU in a clinical sample of Chinese preschool children who had ADHD comorbid with ODD. Furthermore, previous studies of children with ADHD have not measured the correlations between CU traits, EBPs, EF, and the effect of CU traits on the symptoms and diagnosis of ADHD. The incremental validity of the ICU was measured to investigate whether the uitility of the ICU was associated with increasing diagnostic efficacy of ADHD. Furthermore, identifying preschool children who potentially have heightened levels of CU traits is indicative of which children might be at risk of developing ODD or CD.

The current findings are consistent with previous studies [39], which suggests that children with ADHD had a high level of CU traits. CU traits are one of the common psychopathic traits in children with ADHD and ODD. Children with CU traits tend to have more severe and persistent behavioural problems, such as higher levels of aggression. Longitudinal studies of children with ADHD and CU traits demonstrated that approximately 21% developed Antisocial Disorder (ASPD) in adulthood [40–44].

Forthemore, the research team found that compared with ADHD only, children with ADHD + ODD had higher callousness subscale scores. Detecting differences in CU traits in children with ADHD is particularly important to identify possible populations with co-occurring ODD. In this study the callousness traits of ADHD + ODD was similar to previous studies, demonstrating that elevated scores for CU characteristics increase the odds of ODD [31]. Although the current study indicated that children with a co-comorbidity of ADHD + ODD present with significantly higher callousness symptoms than children with ADHD only, the difference was not enough to clearly discriminate children with ADHD and ADHD + ODD in this study, owing to no difference on ICU total score. The children in this study may have been too young to fully present CU traits and ODD symptoms.

The results further revealed that higher levels of CU traits were related to lower levels of prosocial behaviours, such as less consideration of others, sharing with others, and providing help to people in difficulty. Meanwhile, children with high levels of CU traits had more difficulties with EBPs including CPs and peer problems. This finding supports previous evidence in dicating that CU traits are correlated with the severity of behavioural disturbances [45]. More specifically, children with heightened levels of CU traits tend to exhibit high and persistent levels of CPs, inattention and hyperactivity symptoms, impulsivity, and narcissism [16]. CU traits have shown a certain stability from early childhood to adolescence [12, 46], and they predicted the occurrence of CPs and emotional problems, as well as predicting juvenile delinquency [47]. Other studies also demonstrated that CPs early in life are associated with a CU personality [48–50] and a high risk for psychosocial problems [10, 51, 52]. Peer problems usually present as bullying. Previous research has shown that CU traits are viewed as a risk factor for bullying victimization in preschool children [53] and that bullying is related to peer difficulties [54]. Based on the above findings, the researchers could conclude that children with ADHD, especially ADHD + ODD, have higher levels of CU traits during the preschool period. The co-occurrence of ADHD and high levels of CU traits might be the main reasons and indicate a high risk of developing CPs, which is more likely to develop into antisocial personality.

This research also confirmed that CU traits were marginally correlated with EF. These findings are supported by previous studies, which suggest that EF deficits in children with ADHD are conditional upon the level of CU traits [11, 29]. Studies concerning the relationship between EF and CU traits are still scarce and if a point for future research [53]. Across follow-up studies, CU traits have predicted behavioural problems present in late childhood and adolescence [12]. Therefore, more longitudinal studies on CU traits, in early childhood, with later behavioural disorders and cognitive function, need to be carried out in the future.

In terms of the influence of CU traits on ADHD assessment, preliminary analyses demonstrate that CU traits are significantly associated with ADHD symptoms, and it is one of the risk factors for the presence of ADHD. The influence of total score of ICU on ADHD symptoms can reach 15–24%. Moreover, the dimensional analyses indicated that elevated levels of CU traits may also emerge as a predictor for the risk of ADHD in young children when controlling for other possible covariates, such as child sex, CPs, and oppositional defiant symptoms. The incremental predictive utility of ICU showed that the CU traits could be used to explain 6% of the variance in ADHD symptoms. Although this research did reveal that the influence of CU traits was relatively weak as a risk factor for ADHD. Importantly, the researchers further calculated the specificity and sensitivity of the ICU scale in the diagnosis of ADHD, which indicated that the diagnostic value of ICU for ADHD was medium. Taken together, these findings suggest that CU traits are a proposed antecedent of ADHD and might be regarded as an auxiliary diagnosis tool of ADHD.

Some limitations to the present study need to be addressed. Other cross-sectional and longitudinal studies with large samples of preschool children have suggested that when sex, temperament, executive function, and other covariates are controlled, preschool children’s CU traits are effective in predicting the occurrence of CD and ODD [29]. However, due to the low prevalence rate of ODD/CD and the co-occurring rate of CD with ADHD in preschool children, this study did not establish an ODD or CD group to explore the relationship between CU traits, CD, and ODD in preschool children. Therefore, this study only used cross-sectional methods and conducted analyses to explore the effects of CU traits on ADHD symptoms. Based on the findings, it is necessary to follow up and explore the trajectories of CU trait development in children with ADHD from preschool to school-age, and up to adulthood. In future research, different methodologies and analyses could be used to explore developmental heterogeneity in CU traits and the association of these traits as possible with protective factors and risk variables. It remains unclear whether high levels of CU traits predict the forementioned disorders as children with ADHD are more likely to develop ODD or CD in the future.

### Conclusion

There is growing interest in investigating CU traits in ADHD children. The most important insight from this study is that the increase of ICU scores during early childhood tended to be associate with the likelihood of ADHD. These findings provide experimental evidence for the importance of taking ICU into account as an useful assessment tool for ADHD. Identification of early CU traits may help researchers better understand symptoms and behavioural problems in children with ADHD. Early detection may help to implement timely intervention strategies to prevent psychopathy and antisocial personality disorder developing.

## Data Availability

The datasets used and analysed during the current study is available from the corresponding author on reasonable request.

## Appendix 1

See Table 5

**Table 5:** Demographic and clinical characteristics of the study sample

	ADHD (*n* = 89)		TDC (*n* = 87)	F/χ^2^	*p*
	ADHD only (*n* = 55)	ADHD + ODD (*n* = 34)			
Age (months), mean ± SD	56.40 ± 6.66	57.65 ± 9.77	55.16 ± 7.29	1.37	0.26
Sex, n (%)				3.39	0.18
Female	12 (13.5)	20 (23.0)			
Male	77 (86.5)	67 (77.0)			

## Appendix 2

See Table 6

**Table 6:** Comparison of CU traits between boys and girls in ADHD group (mean ± SD)

Subscales	Boys (*n* = 77)	Girls (*n* = 12)	Total (*n* = 89)	*F*	*p*
Callousness	9.71 ± 5.37	8.08 ± 3.80	9.49 ± 5.20	1.022	0.315
Uncaring	16.41 ± 3.92	15.17 ± 4.02	16.24 ± 3.93	1.032	0.312
Unemotional	4.66 ± 2.43	4.08 ± 2.50	4.58 ± 2.43	0.575	0.450
Total ICU	30.66 ± 9.62	27.33 ± 7.04	30.21 ± 9.35	1.321	0.254
